# Acquired Brachial Cutaneous Dyschromatosis: A Rarely Recognized Condition

**DOI:** 10.5826/dpc.1101a111

**Published:** 2021-01-29

**Authors:** Jorge Román-Sainz, Sergio Samer Tabbara-Carrascosa, Marta Martínez-García, Adrián Imbernón-Moya

**Affiliations:** 1 Dermatology Unit, Hospital Universitario Severo Ochoa, Leganés, Spain

**Keywords:** dyschromatosis, poikiloderma, sun damage, hyperpigmentation, pigmentary disorders

## Introduction

Acquired brachial cutaneous dyschromatosis (ABCD) is a pigmentary disorder located on the forearms, especially affecting middle-aged women aged 50–70 with Fitzpatrick’s phototype III–IV [1]. There are only 23 cases reported in the literature, although it is likely underreported by patients due to its lack of symptoms [2].

## Case Presentation

**Figure 1:**
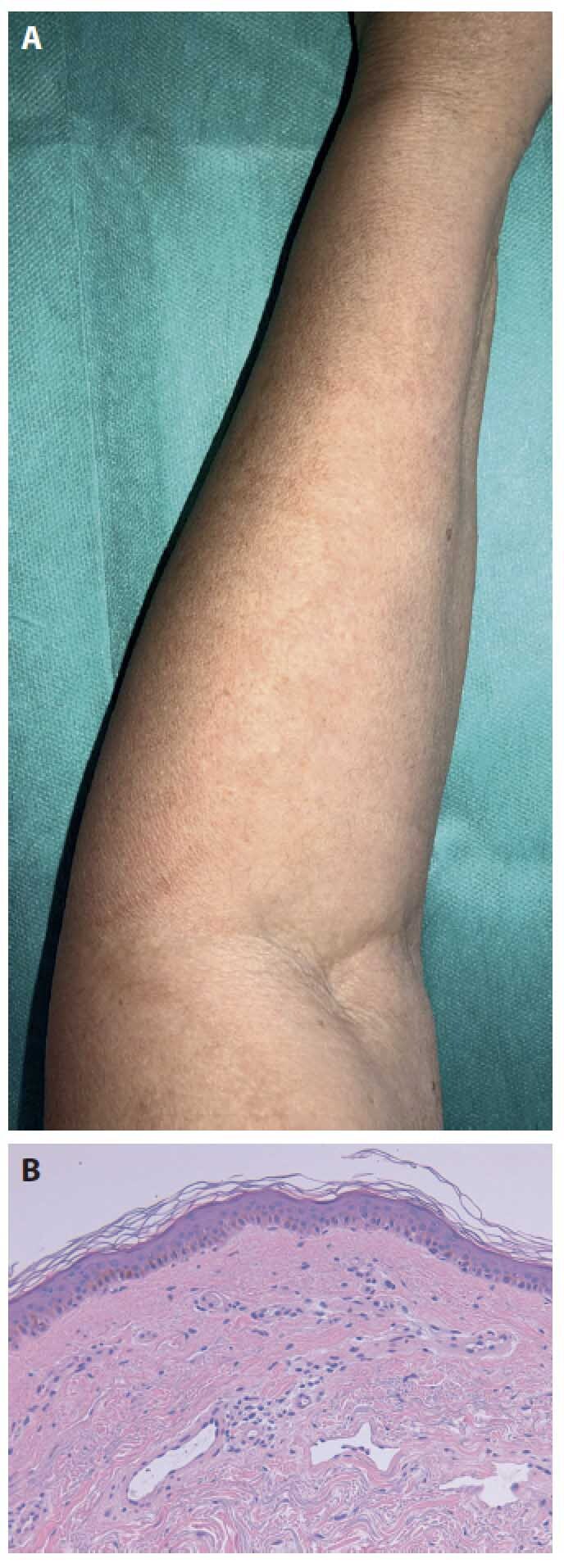
(A) Clinical image showing linear hyperpigmented macules with atrophic and poikilodermal skin. (B) Histopathological image showing epidermal atrophy, as well as hyperpigmentation of the basal layer, solar elastosis, and telangiectases at the superficial dermis.

A 71-year-old woman, with skin type III was referred to the Dermatology Unit for a 6-month history of asymptomatic skin lesions on her forearms. She reported intense, chronic sun exposure. She was being treated with lisinopril, an angiotensin converting enzyme (ACE) inhibitor, due to hypertension. Dermatological exploration showed confluent, symmetrical, light brown hyperpigmented and hypopigmented macules with linear and oval morphology and cutaneous atrophy (Figure 1). Skin biopsy revealed a preserved epidermis with slight hyperpigmentation of the basal layer, and a few telangiectases at the dermal level with marked actinic elastosis. The patient was diagnosed with acquired brachial cutaneous dyschromatosis (ABCD). Photoprotection was prescribed, and she had partial improvement of the lesions after 6 months.

## Conclusions

ABCD was first reported by Rongioletti and Rebora in the year 2000 [1]. It is an acquired pigmentary disorder that presents as asymptomatic light brown patches interspersed with hypopigmented macules [1]. Histology shows poikilo-dermatous tissue with hyperpigmentation of the basal layer, solar elastosis, and superficial telangectases [1,2]. Most of the reported cases present bilaterally on the distal aspect of the forearms [1].

Twenty cases were reported (85%) in females, with only 3 cases in males [1,2]. The age of onset ranged from 50–70, and 91% of cases have been reported in patients with skin phototype III–IV, with only 2 cases being a type II [1,2]. An association with Civatte poikiloderma was found in 9 (39%) patients [1]. The incidence and prevalence of this disorder are unknown. ABCD is thought to be an underdiagnosed disease, likely due to lack of consultation by the patients.

There are currently 2 hypotheses about the etiopathogenesis of ABCD. The first one states that ABCD is related to chronic treatment with ACE inhibitors; 14 (60%) patients were reported to have been in treatment with such drugs for years [1]. The other theory claims that ABCD is more related to cumulative sun damage, as most of the patients had evidence of chronic sun exposure [2]. Both hypotheses could explain the cause of ABCD in a majority of these cases, and this is likely due to an interaction between ACE inhibitors and sun exposure in predisposed patients.

Differential diagnosis must include Civatte poikiloderma, melasma, postinflammatory hyperpigmentation, pigmented contact dermatitis (Berloque dermatitis), macular amyloidosis, prurigo pigmentosa, and drug-induced hyperpigmentation. Although these disorders may be clinically similar, they can be easily differentiated with histopathogical examination.

There is no standard treatment for ABCD. Strict photoprotection is the most widely recommended, but a combination of topical depigmenting agents, chemical peels, and laser treatments can be considered [2].
